# Using an eHealth Intervention to Stimulate Health Behavior for the Prevention of Cognitive Decline in Dutch Adults: A Study Protocol for the Brain Aging Monitor

**DOI:** 10.2196/resprot.4468

**Published:** 2015-11-10

**Authors:** Teun Aalbers, Maria AE Baars, Li Qin, Annet de Lange, Roy PC Kessels, Marcel GM Olde Rikkert

**Affiliations:** ^1^ Department of Geriatric Medicine Radboud University Medical Center Nijmegen Netherlands; ^2^ Radboud Alzheimer Center Radboud University Medical Center Nijmegen Netherlands; ^3^ Institute of Social Sciences HAN University of Applied Sciences Nijmegen Netherlands; ^4^ Department of Human Resource Management Faculty of Economics and Management HAN University of Applied Sciences Nijmegen Netherlands; ^5^ Behavioral Science Institute Faculty of Psychology Radboud University Nijmegen Nijmegen Netherlands; ^6^ Norwegian School of Hotel Mangement University of Stavanger Stavanger Norway; ^7^ Donders Insitute for Brain, Cognition and Behaviour Radboud University Nijmegen Nijmegen Netherlands; ^8^ Department of Medical Psychology Radboud University Medical Center Nijmegen Netherlands

**Keywords:** cognition, healthy lifestyle, eHealth, internet, prevention, applied games, protocol

## Abstract

**Background:**

Internet-delivered intervention programs are an effective way of changing health behavior in an aging population. The same population has an increasing number of people with cognitive decline or cognitive impairments. Modifiable lifestyle risk factors such as physical activity, nutrition, smoking, alcohol consumption, sleep, and stress all influence the probability of developing neurodegenerative diseases such as Alzheimer’s disease.

**Objective:**

This study aims to answer two questions: (1) Is the use of a self-motivated, complex eHealth intervention effective in changing multiple health behaviors related to cognitive aging in Dutch adults in the work force, especially those aged 40 and over? and (2) Does this health behavior change result in healthier cognitive aging patterns and contribute to preventing or delaying future onset of neurodegenerative syndromes?

**Methods:**

The Brain Aging Monitor study uses a quasi-experimental 2-year pre-posttest design. The Brain Aging Monitor is an online, self-motivated lifestyle intervention program. Recruitment is done both in medium to large organizations and in the Dutch general population over the age of 40. The main outcome measure is the relationship between lifestyle change and cognitive aging. The program uses different strategies and modalities such as Web content, email, online newsletters, and online games to aid its users in behavior change. To build self-regulatory skills, the Brain Aging Monitor offers its users goal-setting activities, skill-building activities, and self-monitoring.

**Results:**

Study results are expected to be published in early 2016.

**Conclusions:**

This study will add to the body of evidence on the effectiveness of eHealth intervention programs with the combined use of state-of-the-art applied games and established behavior change techniques. This will lead to new insights on how to use behavior change techniques and theory in multidimensional lifestyle eHealth research, and how these techniques and theories apply when they are used in a setting where no professional back-end is available.

**Trial Registration:**

Nederlands Trial Register: NTR4144; http://www.trialregister.nl/trialreg/admin/rctview.asp?TC=4144 (Archived by WebCite at http://www.webcitation.org/6cZzwZSg3)

## Introduction

Multiple systematic reviews and meta-analyses have shown that Internet-delivered intervention programs aimed at health behavior change can have a positive impact on their respective populations. These effects range from weight loss in obese men and women to moderating alcohol intake patterns, smoking cessation, and adjusting dietary patterns [1-4]. Computer-tailored health programs are complex, long-term programs that are appropriate for targeting multiple behaviors requiring a change in behavioral habits [3,5]. In contrast to results reported by Portnoy et al who showed that increasing age was a negative predictor of program effectiveness, a systematic review by our group on the effectiveness of eHealth interventions in older populations provides evidence that older age cohorts can be reached with eHealth interventions [6]. Within the next few years, the upcoming cohort of older adults will be adapted to a new electronic environment, in contrast to previous cohorts who often lacked computer experience and had limited Internet access. This would facilitate further use of eHealth tools, also for prevention targets in the elderly. With Internet penetration in the Netherlands reaching 94% of all households among the population aged 45-75, more widespread use of eHealth by the elderly is likely.

We use Bennett et al’s definition for Internet interventions as “systematic treatment/prevention programs, usually addressing one or more determinants of health (frequent health behaviors), delivered largely via the Internet (although not necessarily exclusively Web-based), and interfacing with an end user” [7]. These eHealth interventions are characterized by being highly structured, mostly self-guided, interactive, visually rich, and they may provide tailored messaging based on end-user data [2,8]. Additional benefits of Internet programs are their 24-hour availability, uniformity in data dissemination and collection [2,9], and their heightened reach [5]. An advantage of creating such a completely self-motivated eHealth program, in comparison to an expert-led intervention, is the fact that the reduction in needed external support exponentially increases the reach of Internet interventions. Thus, Internet interventions can reach as many participants as is technically allowed by the hosting servers [10]. Moreover, since most of the cost of Web-delivered health programs are associated with the development stages rather than the implementation stage itself (in comparison to regular face-to-face treatment), even programs with relatively low effectiveness but a very large reach, could significantly impact public health [4].

Lifestyle interventions through the Internet are not new. However, online lifestyle intervention programs with cognitive functioning as a primary outcome measure are not yet widespread. The next section presents a short overview of the relationship between six major modifiable lifestyle factors and cognitive functioning.

Physical activity is associated with a lower risk of Alzheimer’s disease or any type of dementia, and older people with better cardiovascular function, who are more physically active, have decreased chances of cognitive decline [11,12]. Already in 2007, a plea was made for physical activity trials as prevention for cognitive decline [13]. Furthermore, physical inactivity has been calculated to account for approximately 5.3 million premature preventable deaths in 2008, effectively decreasing global life expectancy by 0.7 years [14]. The Internet has proven to be a valid way of changing participants’ physical activity levels [15]. Albeit in modest ways, average significant effect sizes of 0.14 [16], 0.16 [1,4], and 0.17 [1] can mean great benefits on a societal level.

Good physical fitness (positively) and higher body mass index (negatively) are related to academic performance as early as in third and fifth grade [17]. These effects seem to transfer to later life, with high blood pressure and central obesity being negatively related to global cognitive functioning in general and more specifically executive functions, visuomotor skills, and memory [18,19]. Although the exact mechanisms and functions that are affected still need to be established by future research, being overweight appears to provide additional risk for cognitive impairment. A recent review summarizes the positive effects of antioxidants and balanced nutrition on the delay and avoidance of onset of dementia [20].

Smoking is one of the most studied health behaviors, but only recently researchers have started to investigate whether smoking cessation has a positive effect on cognitive functioning. Even though results do not yet appear definitive, most research points towards current smoking as a risk factor for Alzheimer’s and vascular dementia [21]. However, smoking cessation seems to mitigate the effects of smoking in the past, and relative risk of getting neurodegenerative diseases later in life decreases to normal levels [21,22]. Depending on the number of cigarettes a person smokes daily, the risk of various forms of dementia increases by 1.59 up to 2.72 times [23-25]. In addition to an increased risk of getting dementia, smokers generally have a lower level of cognitive functioning while smoking and experience faster decline as they age [26].

Alcohol consumption is not an unequivocal area in comparison with the behaviors discussed above. Low to moderate alcohol consumption may very well have positive effects on brain health, but too much alcohol is harmful to the brain. Cross-sectional studies show that moderate alcohol consumption (up to three units a day) may have beneficial effects on episodic memory, executive functioning, and processing speed of the brain [27-30]. However, these results should be interpreted with care. There are no systematic or controlled-trial intervention studies available that examine the influence of alcohol consumption on cognitive functioning, but earlier research has shown that alcohol consumption higher than three units per day is harmful to the brain and can cause Korsakoff’s syndrome [31]. In addition, it is not clear whether the positive effects on cognition are the direct result of the alcohol consumption itself. It may also be that people who have a lifestyle that includes moderate alcohol consumption also moderate themselves in other lifestyle areas, making them better cognitive agers. Further, a recent meta-analysis of epidemiological studies claimed that a reduction of 17% in alcohol consumption causes a 10% reduction in risk of cardiovascular diseases [32].

In a very elaborate review, Goel et al conclude that both acute and chronic sleep deprivation severely influence cognitive capabilities, starting with a measurable drop in performance on executive functioning tasks after being awake for 16 hours [33]. Among others, sleep deprivation further negatively influences psychomotor speed, learning and memory, and working memory performance, and causes faster performance deterioration on longer tasks [33]. Sleep deprivation by lifestyle choice, whether it is chronic or acute, affects executive functioning as the prefrontal and anterior cingulate cortices and posterior parietal systems are especially susceptible to sleep loss [33].

An increase in psychosocial stress can lead to burnout or depression, which negatively affects a person’s cognitive functioning [34]. Among others, attention, concentration, flexibility, and memory deteriorate with higher amounts of perceived stress [19,35]. Epidemiological research shows a connection between the tendency to experience stress and the risk of mild cognitive impairment and Alzheimer’s disease [36-38]. Also, the speed at which older people experience cognitive decline is correlated with the tendency to experience stress [36].

Managing these modifiable lifestyle factors could serve as a strong protective factor against neurodegenerative syndromes such as dementia. Stimulating health behavior change via the Internet appears feasible, and even the use itself of computers may serve as a protective factor when it comes to dementia [39]. Therefore, we plan to design an online, complex eHealth intervention aiming at lifestyle improvement with cognition as the primary outcome measurement. The research question for the current intervention with the Brain Aging Monitor (BAM) will be twofold: (1) Is the use of a self-motivated, complex eHealth intervention effective in changing multiple health behaviors related to cognitive aging in Dutch adults in the work force, especially those aged 40 and over? and (2) Does this health behavior change result in healthier cognitive aging patterns, thereby possibly preventing or delaying future onset of neurodegenerative syndromes like Alzheimer’s disease?

## Methods

The methodology of this study protocol follows the Standard Protocol Items: Recommendations for Interventional Trials (SPIRIT) guidelines that are specifically developed to provide guidance for researchers to document their study protocol and ensure that no relevant information is missing [40].

### Study Design

For this study, we will use a quasi-experimental longitudinal pre-posttest design, with measurements at baseline and after 12 and 24 months. Only Dutch individuals within the Netherlands will be recruited. The intervention website is programmed in Dutch and as such will not be feasible for implementation outside the Netherlands. Since the Brain Aging Monitor is an eHealth intervention and the Netherlands has an Internet penetration of 94% of all households [41], there is no limit to its potential reach within the Dutch-speaking community. There are no regional restrictions that keep uptake of the intervention pinned to the region of the research institute. Since the entire intervention is based on self-management, contact with the research team is strictly limited to technical support. Considering the fact that the protocol relates to a pragmatic field study that will recruit both at an individual and organizational level, we cannot give an accurate estimate of the number of sites needed to obtain the necessary number of participants. For a sample size calculation, we refer to the description of sample size (Nederlands Trial Register: NTR4144).

### Eligibility Criteria

The intervention will be performed among the general Dutch population and is aimed at delaying and/or slowing down cognitive aging. A recent study by Singh-Manoux et al showed that cognitive decline can be measured as early as 45 years of age [42]. For this reason, participants are eligible for analysis of the primary outcome if they are at least 40 years or older. The upper age limit is a more pragmatic one, since the intervention is aimed at the Dutch workforce and 67 is the official retirement age. Apart from this age criterion, a participant has to have sufficient comprehension of the Dutch language to understand the digital informed consent form (see Multimedia Appendix 1) and should have regular access to an Internet connection. Because the entire intervention takes place outside of the research facility, no strict control or enforcement is possible over other ineligibility criteria such as neurodegenerative disorders, medicine use, or psychiatric symptoms. Therefore, we decided not to use these and other possible exclusion criteria, which also increases overall external validity.

### Health Behavior Change Theory

Using Lustria’s organizing heuristic for strategies in computer-tailored online behavioral interventions, the BAM is an iterative, self-guided customized health program, with expert-led technical support [5]. The BAM uses different modalities such as Web content, email, online newsletters, and online games. To build self-regulatory skills, the BAM deploys goal-setting activities, skill-building activities, self-monitoring, and email reminders. According to a meta-analysis by Webb et al, applying a more extensive use of theory in online lifestyle tools increases overall effect size [4]. Theory can aid intervention designers identify appropriate targets for intervention, select intervention techniques, and it can illuminate which mechanisms of change are effective [43]. How theory is applied in the BAM will be described after the description of the intervention program itself.

### Intervention

**Figure 1:**
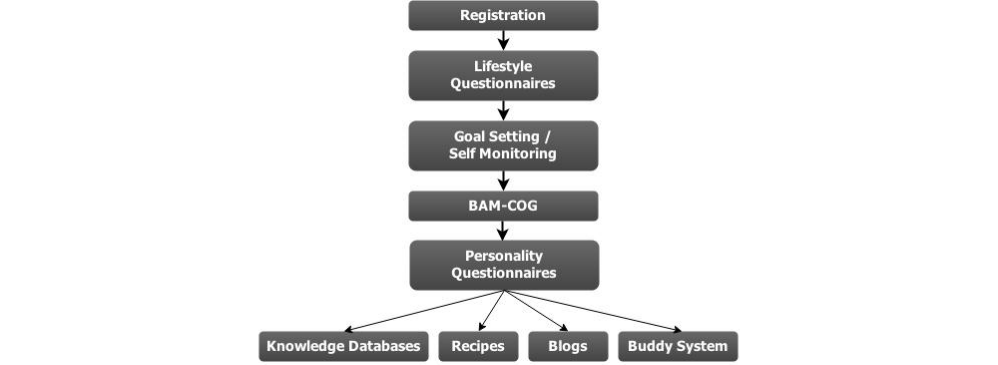
Flowchart of process a new participant goes through upon registration.

**Figure 2:**
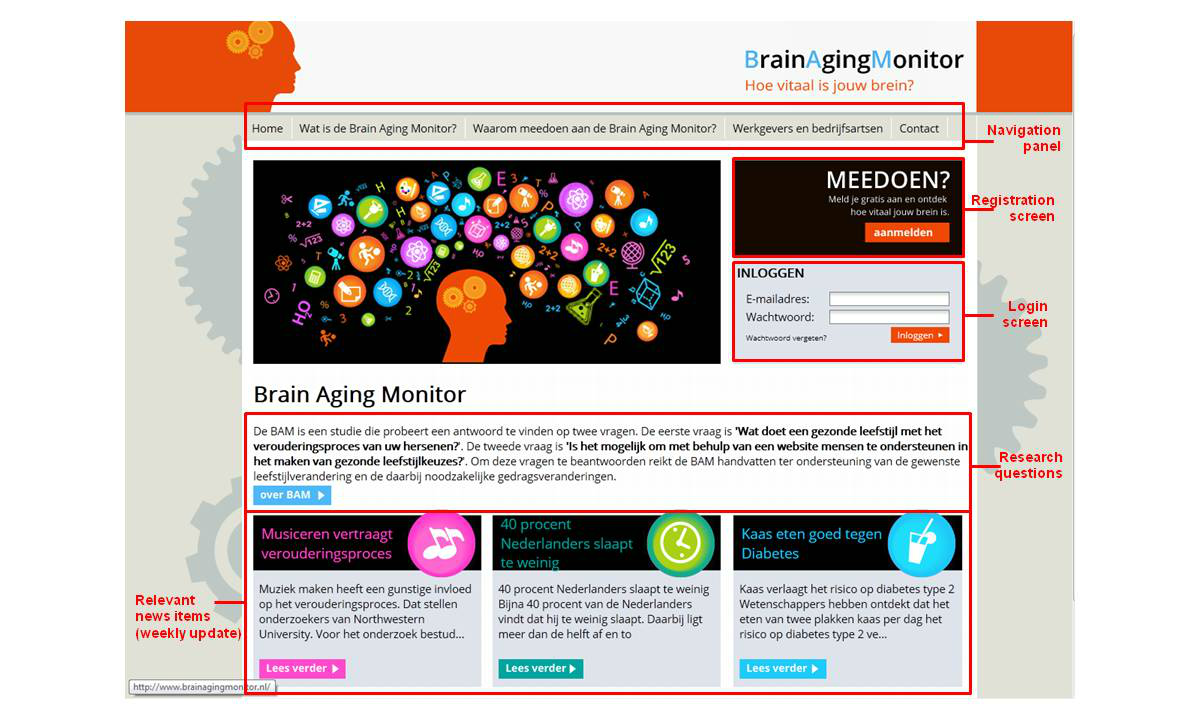
Brain Aging Monitor homepage.

**Figure 3:**
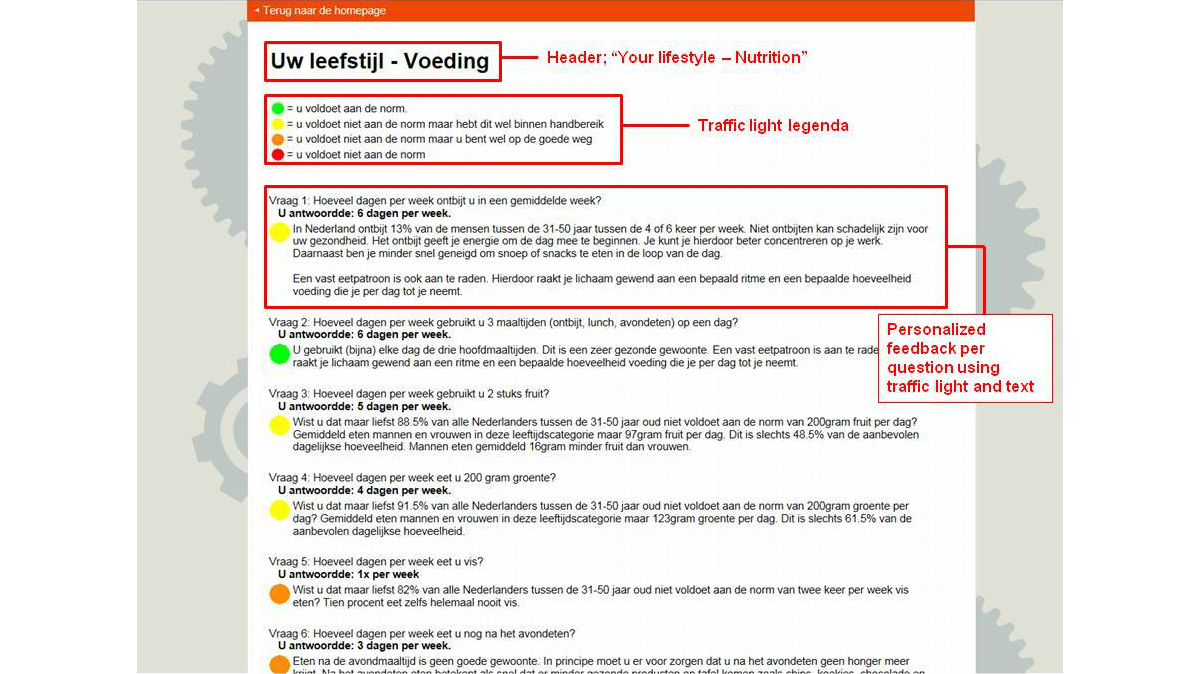
Screenshot of feedback module on nutrition questionnaire.

The BAM eHealth intervention website is open to anyone who is interested in the relationship between healthy living and brain aging. Figure 1 gives a short overview of the flow of a new participant within the intervention, and Figure 2 shows a screenshot of the BAM homepage. The BAM is a complex intervention [44], using multiple intervention components aimed at multiple health behaviors. As mentioned, the BAM focuses on physical activity, nutrition, smoking, alcohol, sleep, and stress. The BAM has an assessment and feedback system. After registering, validating their email address, and signing a digital informed consent form, new participants fill out seven short questionnaires (ranging from 4-20 questions or statements): one questionnaire for every lifestyle factor and one additional questionnaire on individual characteristics. Full lifestyle questionnaires and their references can be found in Multimedia Appendix 2. The answers to these questionnaires are used to create a personal lifestyle profile for the participant. The participant receives feedback per question using an easy-to-understand visual traffic light (green=conform to the norm, yellow=close to the norm, orange=much room for improvement, red=non-norm compliant) based on the health authority recommendations, behavior-specific feedback on health authority recommendations, and reference values on peer behavior (if possible and/or applicable divided for age and gender). Figure 3 shows a screenshot of the feedback on the nutrition questionnaire. This gives the participant a fast and detailed overview of their current lifestyle status. Also, the answers to the questionnaires tailor the intervention to the participant. For example, non-smokers will not be confronted with information about smoking or the option of setting goals that apply only to smokers.

The use of short self-reporting questionnaires on health behavior is a decision from a time-saving and retention perspective. Using more elaborate questionnaires would allow for better insight in a participant’s behavior but will likely result in higher attrition [45]. Furthermore, more elaborate questionnaires are likely to pose questions that are difficult to answer for an individual. For example, obtaining a meaningful, valid answer on a participant’s consumed dietary fiber (using the Dutch Healthy Diet index [46]) is very difficult. This would require a 24-hour recall process during the initial registering procedure, risking immediate dropout. Therefore, we chose to use simple questionnaires for all lifestyle areas, where every question covers a behavioral trait that directly relates to a goal that can be set later on in the program.

After the questionnaires, the Brain Aging Monitor Cognitive Assessment Battery (BAM-COG) opens up on the game wall and the Goal-Setting Module (GSM) is unlocked. The BAM-COG is an online cognitive assessment battery that has been specifically developed for use in the BAM and has been validated by our group [47]. These games measure working memory, visuospatial short-term memory, episodic recognition memory, and planning. An instructional arrow will direct the participants’ attention to the fact that the games are open for play. After receiving their personal lifestyle overview, participants can start setting monthly, personal-health behavior goals using the GSM. We based the GSM on the Goal Attainment Scaling (GAS) methodology by Kiresuk [48]. Using the GAS triggers participants to be more conscious about their goals because it does not rely on a single digit. Instead, it requires the participant to fill out a complete scale from -2 to 2 (where -2=“I have made minimal progress”, 0=“I have reached my original goal”, and 2=“I have done a lot better than my original goal”). This not only requires more attention from the participant while setting the goal, but it also enables the BAM to give positive feedback on partially accomplished goals instead of a “yes” or “no” answer to the question “did you reach your goal?”.

Every potential goal is accompanied with a set of instructions guiding the participant towards personally relevant and realistic goal setting. It starts with an example GAS scale, a case of a fictive participant, and a step-by-step instruction to complete the goal-setting process. The GAS system is programmed to return a number of restrictions or error messages to the user: (1) if values overlap, (2) if values are in the wrong direction or scrambled, (3) if values exceed the value of 7 days per week, or (4) if the given 0-value is a step back from the value that was answered during the intake questionnaire. When the goal is set, the participant is given reinforcing feedback on making a good first step towards behavior change. After this message, a list of instructions and tips are given that are relevant for that specific goal. This list is open for the participant to choose their preferential working method and go from very basic instructions (eg, “buy fruit” in case of a goal “eat more fruit”) to signing social contracts or using implementation intentions (eg, “if there is no running group in the neighborhood, then I will start my own running group” in case of a goal “start to work out”) [49].

After a participant has decided which instructions and tips to use, the goal gets transferred to the short-term monitoring system (STMS). Here, participants can monitor their own behavior on a day-to-day basis. Inputting their behavior in the STMS, the system automatically graphs a quick overview of how well a participant is doing for that goal on a week-to-week basis. After a month, the STMS asks the participant to what extent the goal is accomplished on their own original GAS scale. For any score specific to that goal, the BAM gives tailored feedback. If a score of -2 or -1 is obtained, the goal is deleted from the participant’s profile and encouragement to try again is given. However, if a score of 0, 1, or 2 is obtained, the goal gets transferred to the long-term monitoring system (LTMS). In the LTMS, a participant gets monthly follow-up questions to monitor if they still are maintaining their initial level of behavior change. With every monthly question that gets answered, the participant is given tailored feedback to acknowledge their success or motivate the participant to maintain their initial behavior change. Multiple goals can be graphed over time giving a personal overview of all acquired and maintained behavior change goals. If multiple goals are set on the same subject, new goals will overwrite old goals so that only the most up-to-date information is shown. Because the BAM does not dictate how many a goals a participant sets and in what order they do this, it implicitly provides the participant with a conceptual choice between simultaneous or sequential goal setting, giving every participant the option to work at their own pace and preference [50-52].

In order not to overload the participant with questionnaires immediately after registering, the personality questionnaires become available to the participant 7 days after registration. These are the Dutch General Self-Efficacy Scale [53], lifestyle factor specific self-efficacy questions [54], the Positive Affect Negative Affect Scale [55], and the Self-Control Scale [56]. These questionnaires are administered to perform secondary analyses to check if the BAM is more effective in certain personality types. No feedback on the personality questionnaires is provided for the participants.

After all baseline data are collected, the tailored knowledge databases, the buddy system, healthy recipes, and blogs on health behavior and cognitive aging become available. The buddy system is a built-in control mechanism the BAM uses to ensure goal safety. A subset of goals that are suitable for this purpose are anonymously sent to another BAM user to be judged by a BAM buddy on its feasibility and safety. A buddy can judge a goal to be “ambitious”, “not ambitious enough”, “just right”, or in the case of losing weight, “this seems unhealthy”. This gives the goal-setter an opportunity to get instant feedback on the feasibility of their goal. Also, it gives the buddy a “look behind the scenes” that may provide feedback on their own goal-setting behavior, as the exact same situation for somebody else may be perceived as harmful whereas this same goal would be deemed applicable to the participant themselves. At the same time that the buddy system becomes available, participants also get access to the knowledge databases that contain up-to-date information on healthy living and the relationship between the different behaviors and brain health. Last, participants get access to weekly blogs on lifestyle, research and brain aging, and healthy recipes. After 365 and 730 days upon completion of the baseline measurement, they will be recruited for 1-year and 2-year follow-up measurements.

No reasons for discontinuation of the study of a participant by the research team have been specified. There is no disease load that can be exacerbated by participating in the BAM nor is there any medication prescribed that could have negative health consequences. Participants are provided with the option of unsubscribing to the study at any given time in their personal profile space. When a participant decides to leave the program, a short questionnaire is automatically presented to collect data on the reason for unsubscribing and to inquire if the participant may be approached to partake in the 1-year and 2-year measurement, regardless of subscription status. All of this is voluntary and participants can always choose to skip this questionnaire.

Keeping participants engaged with eHealth intervention programs has been a major problem since the field originated [7,57,58]. Several adherence-enhancing strategies are in place with the BAM. First, we upload weekly news updates on the homepage regarding health behavior and brain aging from the largest Dutch news websites. Second, on the dashboard of the participant we will upload weekly blogs and healthy recipes so that the content, apart from user input, changes on a weekly basis. Blogs discuss current topics in research on anything BAM-related in an easy accessible form. Recipes use fresh and healthy products and provide participants with ideas to prepare a healthy meal. Third, a personalized email reminder system is built into the BAM that can be adjusted to the participants’ individual needs. In their personal profile space, a participant can choose to receive daily, weekly, biweekly, or monthly email reminders. These reminders give an overview of current active goals and will link the participant directly to this goal after logging in. Fourth, during the registration process new participants can opt in to receive BAM newsletters, which will be sent using the MailChimp engine. At any time, participants can opt in or opt out of the newsletter. Last, participants get a personal profile space with a quick and easy overview of their current lifestyle. They can make adaptations to their lifestyle, see the results of their goals, and can adjust their settings.

Considering the field setting for this intervention, it is difficult to control for concomitant care, or better yet if the BAM inspires participants to make use of other platforms to alter their health behavior in a positive way, this accomplishes the BAM’s goals. The BAM can and may function as a gateway to healthy behavior. Using the GSM and the yearly follow-up, the BAM can track changes over time even if participants actively use outside help that the BAM refers them to.

### Use of Health Behavior Change Theory

A description of the behavior change techniques used is given using the taxonomy provided by Abraham and Michie [59]. This paper provides researchers with 26 behavior change techniques based on behavior change theories to be used while designing an intervention. As the BAM is a self-guided, voluntary online intervention, the majority of participants will be in the last three stages of the Transtheoretical Model (TTM): the preparation, action, and maintenance phases [60]. The BAM guides participants from preparation phase (informing) to action phase (goal setting, short-term self-monitoring) to maintenance phase (long-term self-monitoring). As participants enroll in the BAM, the three stages of the TTM are facilitated instantaneously. And for every behavior, the participant can choose the most appropriate phase to start with. When the preparation phase is chosen, the knowledge database for that behavior is the most suitable starting point. Providing a participant with information is in accordance with the Information-Motivation-Behavioral Skills Model (Fisher).

When participants decide to enter the action phase of the TTM and become active goal-setters, they get an appropriate list of instructions and tips. Setting active behavioral goals is a part of most renowned behavior change theories such as the Theory of Planned Behavior (TPB) [61] and the Social Cognitive Theory (SCT) [62]. In line with the SCT, general encouragement is provided when goals are set and (partially) accomplished. While working with the tips and tools, participants will monitor their behavior. Bandura notes in his SCT that self-directed change can be promoted with features that allow program participants to set realistic goals, provide them with instructions and tips to reach these goals, and to allow for detailed self-monitoring of their own behavior over time [63,64]. Using GAS prompts a participant to set specific lifestyle goals, the buddy system prompts the review of behavioral goals, the short-term monitoring system prompts self-monitoring of behavior, and evaluating their own performance gives the participant feedback on their own behavior. The instructions and tips, when of additional value, include a list of commonly heard behavioral barriers and reasons why these barriers are either invalid or on how to overcome them, which is part of the SCT. Another possible instruction was to set up a behavioral contract with a person close to the goal-setter to create a form of peer pressure or control, which is in line with the theory of operant conditioning [59]. Among the instructions and tips, when applicable, is the suggestion to form “if-then” implementation intentions [63]. These if-then implementation intentions partially ease the transformation of behavior but also aid in relapse prevention, as these actively trigger the goal-setter to identify risk situations and come up with an appropriate action if the occasion arises (eg, IF my friends keep asking me to drink beer, THEN I will firmly tell them that I am drinking water this evening). Finally, the goal that can be set to reduce stress and optimize satisfaction with life aims at stress reduction through various methods (eg, yoga, mindfulness).

After the completion of a goal, follow-up is built into the system by automatically re-evaluating changed behavior on a monthly basis. Also, using an automated email reminder system, the BAM aims to maximize adherence to the program, providing follow-up prompts. To summarize, in accordance with the taxonomy by Abraham and Michie, 13 out of 26 behavior change techniques are used in the BAM (#1, 4, 5, 6, 8, 10-13, 16, 18, 23, and 24) [59].

### Research Questions and Outcome Measures

Our primary research questions are the following: (1) Is successful health behavior change related to better cognitive aging patterns over time? Change scores will be calculated by subtracting baseline scores from scores at year 1 and year 2, (2) Does the BAM facilitate health behavior change? (see Multimedia Appendix 3 for the construction of the overall lifestyle score), and (3) Does the BAM facilitate health behavior change in certain specific lifestyle areas better than others?

Our secondary research questions are as follows: (1) Is there a dose-response relationship between the number of goals participants set and the expected amount of health behavior improvement? and (2) Does the BAM increase feelings of self-efficacy in health-related behavior and a change in self-control scores from baseline to 1 and 2 years of intervention, as measured with the Self-Control Scale [56]. In other words, does the BAM increase feelings of being in control of one’s life?

Our primary outcome measures are (1) cognitive change over time, (2) overall lifestyle change over time, and (3) specific lifestyle changes over time. The secondary outcome measures consist of (1) number of goals set, (2) change in self-efficacy, and (3) change in self-control.

### Participant Timeline

The timeline for the BAM is straightforward (see Figure 1). Participants need to register only once. Immediately after registering, email validation, and the informed consent form, the lifestyle questionnaires are available. Once the lifestyle questionnaires have been completed, the BAM-COG becomes available. Seven days after subscription, the personality questionnaires appear in the personal dashboard. These features are all presented again to the participant 365 and 730 days after their baseline completion. The GSM, STMS, and LTMS are continuous processes from the moment they first become available to the participant. After 1 year (365 days), the data will be collected for preliminary secondary outcome analysis. After the 2-year follow-up (730 days), the data will be used for analysis of both primary and secondary outcomes. The nature of the grant requires that the intervention remain online even after data collection is finished for the initial study period and that the BAM remain open to the public after the study is completed. Adaptations to the program can be made at this time, according to study outcomes.

### Sample Size

We aim for a group size of 200 to find a 15% reduction on the risk factors (power calculation based on alpha <.05; power of 0.8; two-tailed; n=166; ±20% dropout).

### Recruitment

Different recruitment strategies will be implemented to reach the necessary sample size. First, we will recruit medium to large commercial or governmental organizations through their human resources department or company employed medical staff. The BAM can provide organizations with a concrete intervention program that can substantiate their health policy. Organizations will be recruited by direct inquiry through telephone calls, emails, and will be targeted during several symposia, workshops, and congresses where the BAM will be presented. Once an organization is recruited, the research team in collaboration with the human resources department will develop a tailored recruitment strategy that maximizes the use of existing communication channels within the organization. These organizations are expected to deliver approximately 50% of all study participants.

Next to organizational recruitment, the BAM will also recruit participants in the general Dutch population. A press release will be issued by the Radboudumc to reach mainstream media to generate national attention for the study. The BAM will be advertised at the website of a cooperative research consortium that draws national attention because researchers from four nationally spread out universities will promote this website. Also, we will present the BAM at national and international health care conferences. We estimate that approximately 50% of all study participants will result from this free recruitment strategy.

The enrollment period will take approximately 4-5 months. However, due to the nature of the grant no actual stop in participant influx will be enforced. Participants will be allowed to enter the study at any given time. We will monitor participant influx over time so we can keep estimating the relevance and need for an intervention such as the BAM. Unless individual organizations determine otherwise, no financial incentives will be offered to potential participants. If this occurs, this will be disclosed in future publications.

### Sequence Generation, Allocation Concealment Mechanism, Implementation, and Blinding

This pragmatic field trial does not use a control group. Therefore, sequence generation, allocation concealment, implementation, and blinding are not discussed.

### Data Collection Methods

Data collection in the BAM is completely automated through its website. Therefore training of personnel is irrelevant. All data collection forms will be equal for each new participant who subscribes to the program. For collection of the descriptive lifestyle data, questionnaires have been used that accurately represent the relevant health norm or health behavior (full lifestyle questionnaires can be found in Multimedia Appendix 2). For measuring cognitive functioning, the BAM program uses a validated online self-monitor for cognitive functioning, specifically developed for use in the BAM, called the BAM-COG [47]. We have deliberately chosen to keep the baseline assessment as concise as possible. Creating a complete overview of a participant’s lifestyle can be a tedious task and with high risk of early dropout in eHealth interventions, the BAM’s lifestyle assessment is meant to give a fast and easy overview of a participant’s compliance to health norms, not a detailed description of all facets that make up healthy living.

The BAM intervention has multiple built-in mechanisms aimed at increasing retention to protocol. As described in the intervention section of this protocol, we will use blogs and recipes in the intervention to keep participants’ focus on the program, as well as the deployment of the adjustable reminder system and the flexible use of newsletters. When the program has been online for 365 and 730 days, special newsletters will be sent out to all active participants reminding them of their annual follow-up measurement that becomes available on their personal dashboard.

Participants who want to exit the study can do so at any time. They can unsubscribe from their personal profile page using the unsubscribe instructions. Once this process is initiated by the participant, a short questionnaire will be used to identify the reason for dropout. Also, the BAM will ask the participant if they are still willing to be reminded of the annual measurements. In this case, they would not actively participate in goal setting and behavior monitoring but would be willing to come back and provide the program with follow-up data.

### Data Management

Because of the eHealth nature of the intervention, all data entry and collection are done online and therefore are programmed to be completely automated. The intervention website is secured with up-to-date online security protocols and certificates safeguarding private information of participants. Users must sign in to get access to their profile and logged data. To sign in, a user name (email address) and password are required. Passwords are stored by using the MD5 hash algorithm. Each user gets their own session after signing in. This session will be killed when the user closes the browser or when the session times out. All data are stored in a MySQL database. To access this database, a password is required that contains digits as well as characters, randomly created. The site uses the HTTPS protocol and is secured by a Comodo SSL Certificate.

Data storage will be extensively tested in the pilot phase. All the participants are assigned an anonymous personal identifier that will be used for all the tables containing data during data collection in the MySQL database. The data will be stored on secure hosting servers for 20 years after the completion of the intervention period.

### Statistical Analyses

#### Intervention Effects

Primary analyses will be unadjusted. Depending on the distribution of continuous, categorical, and interval outcomes, an appropriate distribution and relevant statistical models will be used. These models will assess intervention effects at end-of-intervention (1-year and 2-year) as well as the difference between 1- and 2-year measurements. Baseline characteristics will be compared between groups using *t* tests for continuous variables and chi-square tests for categorical variables. Mann-Whitney tests will be used when baseline data are not normally distributed. If necessary, multivariate analysis, including multi-analysis of variance and multiple regression, will be performed to adjust potential confounders, including baseline demographic and behavioral characteristics. The covariates associated with outcomes or contributing to a significant part of variation of used multivariate models will be adjusted as potential confounders. The final model will include these covariates or remove those that do not affect estimates, if models show evidence of overfitting.

#### Secondary Analyses

Total set goals and specific lifestyle area goals will be reported descriptively. The changes of lifestyle within the goal setting group will be reported in absolute difference at end of intervention. The dose-response association between goals and change of lifestyle will be analyzed by multivariable linear regression model, and potential confounders will be adjusted. The change in self-efficacy and self-control scores will be reported in absolute difference at the end of the intervention. The association between the use of BAM and increased feelings of self-efficacy or self-control will be analyzed by multivariate analysis.

Analysis will be performed per protocol. The dropout in this pragmatic field study is likely to be high but can be considered a separate outcome for the implementation and feasibility of these types of intervention. As such, it represents valuable data about the quasi-experimental study design.

### Data Monitoring

There will be no data monitoring committee for this intervention, as no adverse events are expected. No interim analysis will be performed for the same reason. No significant harm to the participants is to be expected for the BAM intervention program. If anything would occur, participants can contact the research team through the contact form on the website. Standard data monitoring procedures for the scientific Geriatric Medicine department at the Radboudumc apply.

### Auditing

No auditing is planned specifically aimed at the BAM study. However, the BAM is part of the scientific branch of the Geriatric Department of the Radboudumc and therefore can be routinely audited internally.

### Research Ethics Approval and Protocol Amendments

The program is largely implemented in medium-to-large corporations that transparently implement the program as part of their health policy. From individual participants, an online informed consent form for their participation in scientific analysis is obtained during registration for the program. This study was deemed exempt from formal ethical evaluation by the local medical ethics committee (region Arnhem-Nijmegen, registration number: 2014-1268). Protocol amendments will be submitted, if necessary.

### Informed Consent

Due to the online nature of this study, no personal informed consent can be obtained from participants. However, we do use an online informed consent form to make sure that participants are aware of their participation in scientific research. Therefore an extra step has been added to the registration process. After email verification, before a participant can start the program, a screen appears with an informed consent form. If informed consent is not provided by ticking the correct box, participation in the program cannot continue. See Multimedia Appendix 1 for a complete translation of the information provided and accompanying informed consent form.

### Confidentiality

All information stored in online databases is random-password protected. Also, all our websites use state-of-the-art SSL-security certificates to ensure maximum safety of participants’ confidential information. MySQL data that contain names are stored in different tables as study results. Exported data from the MySQL online databases will be downloaded to local password-protected hard drives for analysis. Also, when databases are saved on local hard drives, these databases will be stored anonymously, using only anonymous personal identifier codes for all participants. No print records will be kept at any point during the study. Participants’ study information will not be released outside of the study without the documented permission of the participant.

### Access to Data

Only principal investigators, post-docs, and PhD students involved in the study will have access to the full raw dataset. Other researchers interested in using the BAM dataset will get access to a cleaned dataset. Human resources departments of recruited companies will, at no point, get access to any form of dataset. They will receive anonymous overall group results of data analysis.

### Ancillary and Post Trial Care

Participants can always contact the research team by phone or email with any questions they may have. Also, the BAM will remain available to them after the study closes since the BAM is part of a national Quick Results grant aimed at providing fully functional end products at the end of the study period.

### Dissemination Policy

Study results, regardless of their direction of outcome, will be published in high standard, peer-reviewed scientific journals. Study publications will be written on primary and secondary outcomes and subgroup analysis. Researchers and health care professionals will be updated on study results during national and international conferences and workshops and with targeted tailored publications in relevant professional magazines.

Participants who unsubscribe to the program are given the option to stay updated on study results when these become publicly available in the form of a summary of the research report or PhD thesis. Participants who stay in the program until the study period closes will be approached by email to probe their interest in study results. Furthermore, study results will be made available to the general public via a press release issued by the Radboudumc after publication of the primary outcomes. No publication restrictions apply, and no ghost- or professional medical writers are involved in the study.

## Results

Study results are expected to be published early in 2016.

## Discussion

### Principal Considerations

To our knowledge, this is the first large study that aims at health behavior gains with a cognitive motivation and outcome measure in the general population. Furthermore, the BAM is one of the first studies launched in an era when almost everybody has an Internet connection. As such it can serve as a proxy for the feasibility of these types of interventions when specifically launched in the general population.

Enhancing the BAM with state-of-the-art, scientifically validated applied games gives the unique advantage of being capable of measuring cognitive functioning while maintaining all the advantages such as reach and low cost that are associated with eHealth studies. The use of online applied games from the safety and comfort of one’s home gives us another major advantage. It also provides a motivational edge, since playing games is considered more appealing than participating in standard neuropsychological testing. We decided to use self-reporting measures for the BAM instead of more objective clinical measures (eg, blood pressure, cholesterol) because use of these measures in a general community dwelling research population is either not feasible or expensive and a big logistic challenge. Moreover, using a participant’s self-reported input closely matches the participant’s perception of their own behavior. Therefore, the goals a participant sets are more likely to be perceived as personally relevant and the participant will feel ownership over the goal and behavior change that needs to be achieved. Combined, we feel this increases the odds of successful implementation of the BAM.

We chose a quasi-experimental design as it seems more appropriate for a field setting in which it is highly impractical to initiate a randomized controlled trial, and blinding participants to the type of eHealth intervention they are receiving is practically impossible [65]. Theoretically, it was preferable to use a step-wedge cluster-randomized controlled design, but this was not feasible with the current 2-year intervention period. Since recruitment of companies is not guaranteed, cluster randomizing from the start is also difficult, especially since organizations are not very likely to see the incentive of participating as a control group. There is also a pragmatic side to the choice of the population. There is substantial theoretical background to select participants aged 40 and older [42], since in this part of the working population cognitive decline can already be measured, and they are more likely to be triggered by a dementia prevention program. People under 40 are less likely to be triggered by cognitive decline or even dementia prevention, as it is a disease associated with old age and only in later years a relevant threat to their health. Nonetheless, the BAM will allow participants under 40 to subscribe. However, lifestyle advice will be tailored to age cohorts starting at age 40.

Last, the use of a multimodal lifestyle perspective is a strength, as it gives potential participants a more integral overview of lifestyle. Providing a more comprehensive lifestyle overview allows the participant to prioritize one type of change over another and take a holistic approach to their own lifestyle. Also, benefits from changing one behavior may transfer to improved outcomes on another behavior that would go unnoticed in single modal interventions. Since the BAM is an eHealth intervention, tailoring to the needs of the participant is cheap after initial development costs have been incurred. Targeting personally relevant lifestyle factors after providing a more general overview should improve program adherence because the participant becomes aware of why they are working on a certain risk factor. This is important as adherence is often the crucial factor in lifestyle improvement programs.

### Conclusion

This study will add to the body of evidence on the effectiveness of eHealth intervention programs with the combined use of state-of-the-art applied games and established behavior change techniques. This will lead to new insights on how to use behavior change techniques and theory in multidimensional lifestyle eHealth research, and how these techniques and theories apply when they are used in a setting where no professional back-end is available.

## Appendix

Multimedia Appendix 1 Translated version of the informed consent form.

Multimedia Appendix 2 Questionnaires.

Multimedia Appendix 3 Construction of the overall lifestyle score.

## Abbreviations

BAM: Brain Aging Monitor
BAM-COG: Brain Aging Monitor – Cognitive Assessment Battery
GAS: Goal Attainment Scaling
GSM: Goal-Setting Module
LTMS: Long-Term Monitoring System
SCT: Social Cognitive Theory
SPIRIT: Standard Protocol Items: Recommendations for Interventional Trials
STMS: Short-Term Monitoring System
TPB: Theory of Planned Behavior
TTM: Transtheoretical Model
