# Supplementary material for: Using an eHealth Intervention to Stimulate Health Behavior for the Prevention of Cognitive Decline in Dutch Adults: A Study Protocol for the Brain Aging Monitor
Source: JMIR Res Protoc. 2015 Nov 10;4(4):e130. doi: 10.2196/resprot.4468 (PMC4704922; doi:10.2196/resprot.4468)
Supplement: Multimedia Appendix 1 [file resprot_v4i4e130_app1.pdf]

Multimedia Appendix 1. Translated version of the Informed Consent form

Informed Consent form

The Brain Aging Monitor is a study about the brain of the Radboudumc in Nijmegen. The results of the research will be used to learn more about the workings of the brain.

We therefore ask you to agree that we use your answers to the questionnaires and measurement results. If you agree, you can participate in the Brain Aging Monitor program. Before you agree, you must have read the written information about what the research entails. This information is provided below:

What is the Brain Aging Monitor ?

The Brain Aging Monitor is a lifestyle program developed by the Radboudumc. The Brain Aging Monitor (BAM) gives you insight in your lifestyle, makes you more aware of your behavior and supports you in making healthy choices. The BAM supports you in acquiring and maintaining healthy behaviors. For example, exercise, diet , alcohol consumption and smoking . But also sleep and relaxation.

The BAM challenges participants to live healthy and responsible. You will gain insight in your own lifestyle. The BAM compares your personal lifestyle to the standards for healthy living by the World Health Organization and the National Food Centre. Based on these results you set personal and

achievable monthly goals. The BAM provides you with a number of tools that make it easier to achieve your goals. For example, the BAM uses monitoring systems for the amount of exercise performed or the number of units of alcohol you have drunk. On the basis of this information, the BAM gives you insight into your progress to reach your goals.

In order to combine the performance of your brain with these lifestyle changes, the Radboudumc has specially developed a new online self monitor for cognitive performance for the BAM. We developed a new way of using online puzzles. Thus, the BAM can map your memory and planning ability over the course of time. When we link the data about healthy living and your brain aging we can draw conclusions about how healthy living affects brain aging.

The BAM is the subject of Teun Aalbers' PhD thesis. Aalbers is a PhD at the Department of Geriatric Medicine at the Radboudumc. For his study he tries to answer two questions. The first question is "What is the effect of a healthy lifestyle on the aging process of our brain?”. The second question is "Is it possible to support participants in making healthy lifestyle choices using a standalone website?”. The BAM therefore aims to follow you for two years. This may seem very long at first. But breaking unhealthy habits and patterns is not easy. Especially since we’re interested in observing if health behavior changes persist over time and become new healthy habits we need to follow you for a longer period of time. Furthermore, the decline of cognitive functions during our lifespan is a slow and gradual process. To see if making healthy behavioral choices influences this gradual decline the follow up period needs to be several years.

In order to optimally do this the BAM administers a number of questionnaires. These questionnaires are about your current lifestyle. In addition, the BAM asks you a number of

questions that maps your perspective on behavior change and how you cope with difficult situations. This way, over the course of time, we can see if participants who deal with difficult situations in different ways also change their behavior in different ways.

The BAM also asks you to play four puzzle games. This allows us to globally map the performance of your brain. By repeating these games after one year and two years we see how and if you have changed.
We hope that you continue to take part in the study for the duration of at least two years. However, you are free to stop your participation in the study at any given moment in time without providing an explanation. This can be done by clicking the "unsubscribe" button on your personal profile. After this you will receive no further information from the Brain Aging Monitor.

Your information will be kept strictly confidential by the BAM and Radboudumc . Your personal information will not be provided or released to any third party under any circumstances. All your data is treated and analyzed anonymously and will in no way be traced back to your person. If you participate in the BAM through your employer, your employer will not receive feedback on your results that can be traced to your person. Anonymous results will only be provided when there are sufficient participants from the same employer participating to guarantee anonymity.

If, for whatever reason, you are under the care or supervision of a physician, dietitian, physical therapist or other health care professional, you need to follow the advice you have been given by this health care professional at all times. If the advice of the BAM are contradictory to the advice you receive from your therapist, you can discuss this with said health care professional. The advice of

your therapist, however, is completely customized to your situation. So always follow the advice of your health care professional over advice by the BAM.

1. I have read the written information concerning the study
2. I am satisfactorily informed about the study
3. I have had time to think about my participation in the study
4. I know that participation is completely voluntary. I know I can decide not to participate at any given moment in time. I don't need to provide a reason for my discontinuation of the study.
5. I give the administrators of the Brain Aging Monitor permission to access my account when I request assistance.

I hereby consent to the use of my data for scientific purposes, provided that my privacy is guaranteed:

☐ Yes, I have read the informed consent of the Brain Aging Monitor and agree to these terms.
