# Supplementary material for: Using an eHealth Intervention to Stimulate Health Behavior for the Prevention of Cognitive Decline in Dutch Adults: A Study Protocol for the Brain Aging Monitor
Source: JMIR Res Protoc. 2015 Nov 10;4(4):e130. doi: 10.2196/resprot.4468 (PMC4704922; doi:10.2196/resprot.4468)
Supplement: Multimedia Appendix 2 [file resprot_v4i4e130_app2.pdf]

Multimedia Appendix 2. Questionnaires

1. **Demographics**

1. What is your age?

____ years old

2. Gender:

Male / Female

3. What is the highest education you have enrolled in?

answers provided in Dutch as our system differs from the English or US system, ranked from low to high, starting with 'no formal education' up to 'university'

Geen opleiding gevolgd / afgemaakt

Basisonderwijs

MAVO

Voorbereidend beroepsonderwijs

HAVO / VWO

Middelbaar beroepsonderwijs

Hoger beroepsonderwijs

Wetenschappelijk onderwijs

4. Height:

approximately ____ cm

5. Weight: (preferably today or otherwise a recent measurement)

approximately ____ kg

6. How would you describe your general health?

Not good

Moderate

OK

Very good

Excellent

7. Do you find yourself forgetful?

Yes

No

8. How much do you worry about your forgetfulness?

Not at all

Very little

A little

A lot

Very much

9. How many hours per week do you work under contract?

____hours

10. How much overtime do you work on a weekly basis? (Please include both paid and unpaid overtime but at home or at work. Exclude time commuting)

    ____ hours

11. How many hours of your total working hours do you spend behind a computer?

    ____ hours

2. Lifestyle questionnaires

2.1 Physical activity:

The next question is about physical activity like walking, or cycling, gardening, working out or other physical activity at school/work, around the house or during leisure time. Please include all physical activities that are at least as intense as walking briskly or cycling.

1. How many days a week do you at least perform 30 minutes of this type of physical activity? Please consider the average number of days of an average week in the last month.

    _____days per week

The next question is about intense physical activity that noticeably raises your heart rate, respiration and lasts long enough to start perspiring, like exercise or other intense activities at school/work, around the house or during leisure time.

2. How many times per week do you take part in intense exercise or heavy physical activities that last long enough to start perspiring? Please consider physical activity that lasts longer than 20 minutes per bout. Consider an average week in the last month.

_____days per week

3. Do you know the Dutch Norm for Healthy Physical Activity?

- Yes
- No

4. According to you, how much physical activity is necessary to increase your health status? This means something different from maintaining your health status.

- 1 time per week 10 minutes of moderate intense activity, like brisk walking or cycling (15 km/hr)
- 3 times per week 10 minutes of moderate intense activity, like brisk walking or cycling (15 km/hr)
- At least 5 times per week, but preferably daily, 30 minutes of moderate intense activity, like brisk walking or cycling (15 km/hr)
- 3 times per week at least 20 to 30 minutes of intense physical activity like running

5. According to you, how fit are you on a scale from 1-10?

1 2 3 4 5 6 7 8 9 10

2.2 Nutrition:

1. On an average week, how many days per week do you have breakfast?

____ days per week

2. How many days per week do you have 3 regular meals (breakfast, lunch, dinner) a day?

____ days per week

3. How many days per week do you eat 2 portions of fruit?

____ days per week

4. How many days per week do you eat 200 grams of vegetables?

____ days per week

5. How many days per week do you eat fish?

- None
- 1x per week
- 2x per week or more

6. How many days per week do you eat after you had dinner?

____ days per week

7. How many days per week do you have unhealthy snacks in between the three main meals?

____ days per week

2.3 Smoking:

1. Are you a smoker?

- Yes, I currently am a smoker
- No, I have quit in the last six months
- No, I have quit longer than six months ago
- No, I have never smoked

Applicable for smokers only:

2. During last year, how many times did you quit smoking for a period longer than 24hours?

   ___ times

3. Are you seriously considering quitting smoking?

- Yes, I consider quitting smoking within the next 30 days
- Yes, I consider quitting smoking within the next 6 months
- No, I don’t considers quitting smoking at the moment

4. How much do you smoke on a day to day basis? (multiple options possible)

- Less than 10 cigarettes
- 10-20 cigarettes
- More than 20 cigarettes
- Less than 5 cigars/pipes
- 5-10 cigars/pipes
- More than 10 cigars/pipes

2.4 Alcohol:

1. How many days per week do you consume alcohol?

- 0 (go to 2.5)
- 1
- 2
- 3
- 4
- 5
- 6
- 7

2. How many standard drinks of alcohol do you have on a typical day that you consume alcohol?

- 1 glass
- 2 glasses
- 3-5 glasses
- 6 glasses or more

3. On the day of the week that you drink most alcohol, how many standard drinks of alcohol do you consume?

- 1 glass
- 2 glasses
- 3-5 glasses
- 6 glasses or more

4. How many standard drinks of alcohol do you consume weekly?

- 1-5
- 6-10
- 11-15
- >15

2.5 Sleep:

1. On average, how many hours do you sleep per night? Don't count the hours you spent in bed but those that you're actually asleep.

   ………………………………………. hours

2. On average, how many hours of sleep do you think you need per night to function properly the next morning?

   ………………………………………. hours

3. At what time do you normally go to bed?

   ______

4. At what time would you preferably go to bed on a normal night?

   ______

5. On average, how long does it take you to fall asleep?

   ______

6. On average, at what time do you wake up in the morning?

   ______

7. At what time would you preferably wake up on a normal morning?

______

8. How many times do you wake up during the night?

____ times

The following short questionnaire are on your sleep during the night and attentiveness during the day time. Please indicate for every statement what is most appropriate for your situation when you look back at the last four weeks.

0 = never

1 = sometimes, but less than 3 times per week

2 = often, more than 3 times per week

3 = daily

**Situation during the last 4 weeks**	Never	Sometimes	Often	Daily
1 Do you have trouble getting to sleep?	0	1	2	3
2 Do you wake up multiple times during the night and have trouble getting back to sleep?	0	1	2	3
3 Do you experience tension and stress while trying to fall asleep?	0	1	2	3
4 Thoughts are racing through your head while you are in bed?	0	1	2	3

5 Are your muscles tense while trying to fall asleep?	0	1	2	3
6 Do you have the feeling that you sleep restlessness?	0	1	2	3
7 Do you snore?	0	1	2	3
8 Do you experience pain or cramps during the day and/or night time?	0	1	2	3
9 Do you wake up with a headache or painful muscles?	0	1	2	3
10 Do you get enough sleep to wake up refreshed?	0	1	2	3
11 During the day, do you feel drowsy or sleepy?	0	1	2	3
12 Do you take naps longer than 5 minutes during the day?	0	1	2	3

2.6 Relaxing:

1. Satisfaction with Life Scale[66]:

Diener E, Emmons RA, Larsen RJ, Griffin S: **The Satisfaction With Life Scale**. *J Pers Assess* 1985, **49**(1):71-75.

2. Can you estimate how many hours per week you spend on the following activities during a regular week?

Watch TV	___ hours per week
Reading; books, magazines, newspapers, etc	___ hours per week
Being part of a group / clubs	___ hours per week
Sports: ball sports, endurance training etc	___ hours per week
Light sports: hiking, cycling, gardening, etc	___ hours per week

Think sports: checkers, chess, puzzles, etc ___ hours per week

Doing groceries, cooking, physical hygiene ___ hours per week

Hobbies, making music, driving ___ hours per week

Learning new skills: language, courses, etc ___ hours per week

Social gathering with friends, family, acquaintances ___ hours per week

2.7 Personality Questionnaires

2.7.1 Dutch General Self Efficacy Scale:

Schwarzer RJ, M.: **Generalized Self-Efficacy scale**. In: *Measures in health psychology: A user's portfolio Causal and control beliefs.* edn. Edited by J. Weinman SW, & M. Johnston. Windsor, England: NFER-NELSON; 1995: 35-37.

2.7.2 Self Efficacy per lifestyle area:

Kelly RB, Zyzanski SJ, Alemagno SA: **Prediction of motivation and behavior change following health promotion: role of health beliefs, social support, and self-efficacy**. *Social Science & Medicine* 1991, **32**(3):311-320.

2.7.3 The Positive and Negative Affect Schedule (PANAS):

Watson D, Clark LA, Tellegen A: **Development and validation of brief measures of positive and negative affect: the PANAS scales**. *J Pers Soc Psychol* 1988, **54**(6):1063-1070.

2.7.4 Self-control scale by Tangney:

Tangney JP, Baumeister RF, Boone AL: **High self-control predicts good adjustment, less pathology, better grades, and interpersonal success**. *J Pers* 2004, **72**(2):271-324.
