# Supplementary material for: Using an eHealth Intervention to Stimulate Health Behavior for the Prevention of Cognitive Decline in Dutch Adults: A Study Protocol for the Brain Aging Monitor
Source: JMIR Res Protoc. 2015 Nov 10;4(4):e130. doi: 10.2196/resprot.4468 (PMC4704922; doi:10.2196/resprot.4468)
Supplement: Multimedia Appendix 3 [file resprot_v4i4e130_app3.pdf]

Multimedia Appendix 3. Construction of the overall lifestyle score

Lifestyle area	Health Norm Division	Score
Physical Activity	Not active (0-2 days of physical activity per week)	1
	Sub active (3-4 days of physical activity per week)	2
	Norm active (5-7 days of physical activity per week)	3
Exercise	Not active (0 days of exercise per week)	1
	Suboptimal active (1 day of exercise per week)	2
	Norm active (≥2 days of exercise per week)	3
Healthy Nutrition Behavior	Lowest tertile of Health Nutrition scores	1
	Middle tertile of Health Nutrition scores	2
	Highest tertile of Health Nutrition scores	3
Unhealthy Nutrition Behavior	Lowest tertile of Unhealthy Nutrition scores	1
	Middle tertile of Unhealthy Nutrition scores	2
	Highest tertile of Unhealthy Nutrition scores	3
Smoking status	Current smoker	1
	Ex-smoker	2
	Non-smoker	3
Alcohol consumption status	Frequent drinker (≥6 days per week)	1
	Abstainer	2
	Normal drinker (≤5 days per week)	3
Sleep status	Unhealthy sleeper	1
	Suboptimal sleeper	2
	Healthy sleeper	3
Stress status	Lowest tertile of Satisfaction with Life scores	1
	Middle tertile of Satisfaction with Life scores	2
	Highest tertile of Satisfaction with Life scores	3
